# Anterior mediastinal collision tumor of type AB thymoma and adenocarcinoma: a case report

**DOI:** 10.3389/fonc.2025.1495627

**Published:** 2025-05-16

**Authors:** Zhongqin Kang, Rong Xu, Weiya Wang, Keyu Yan, Yi Liao, Lizhi Zhang

**Affiliations:** ^1^ Departments of Radiology, Luxian People’s Hospital, Luzhou, Sichuan, China; ^2^ Departments of Radiology, West China Second Hospital of Sichuan University, Chengdu, Sichuan, China; ^3^ Departments of Pathology, West China Hospital of Sichuan University, Chengdu, Sichuan, China; ^4^ Departments of Pathology, Luxian People’s Hospital, Luzhou, Sichuan, China; ^5^ Departments of Radiology, West China Hospital of Sichuan University, Chengdu, Sichuan, China

**Keywords:** collision tumor, foregut cyst, type AB thymoma, adenocarcinoma, computed tomography

## Abstract

Collision tumors are defined as the coexistence of two or more histologically distinct tumor types at the same anatomical site. Mediastinal collision tumors constitute an exceptionally rare subtype of these lesions. Clinically and radiologically, the preoperative diagnosis is particularly difficult due to their complex pathological composition and nonspecific manifestations. Misdiagnoses are common in clinical practice, even with preoperative needle biopsy. Radiologic identification of collision tumors is essential to ensure comprehensive sampling for guiding appropriate treatments. Herein, we report an extremely rare case of mediastinal collision tumor diagnosed by contrast-enhanced chest computed tomography (CT), with pathology confirming type AB thymoma and well-differentiated adenocarcinoma (likely of malignant foregut cyst origin). This case report aims to raise awareness of this rare tumor and to discuss the imaging features based on a systematic review of the literature.

## Introduction

Collision tumors represent a rare condition in which neoplasms of different histologic origins coexist within the same organ, attached at their margins but without significant intermingling of cellular components. The different components of a collision tumor are anatomically separated from each other, usually by a thin stromal layer or their respective basement membranes (1). Collision tumors have been reported in various organs, including the esophagus (2–4), stomach (5, 6), larynx (7, 8), kidneys (9–11), intracranial region (12, 13), intraocular region (14), pancreas (15), bladder (16), ovaries (17), uterus (18) and other organs. Most collision tumors occur in the digestive or endocrine organs, whereas those located in the mediastinum are exceedingly rare. Mediastinal collision tumors are frequently misdiagnosed or underdiagnosed in clinical practice due to the differing imaging manifestations caused by the independent and primary nature of the tumor components, as well as their adjoining and mixed locations. However, CT serves as an essential tool for diagnosing and differentiating mediastinal collision tumors by precisely demonstrating the tumor location, dimensions, margin characteristics, adjacency, lymph node metastasis, tumor infiltration and providing comprehensive diagnostic information for clinical practice. Here, we present the clinical, radiological, and histopathological characteristics of a rare mediastinal collision tumor to improve recognition of this entity and to guide clinical management and postoperative follow-up.

## Case description

**Figure 1:**
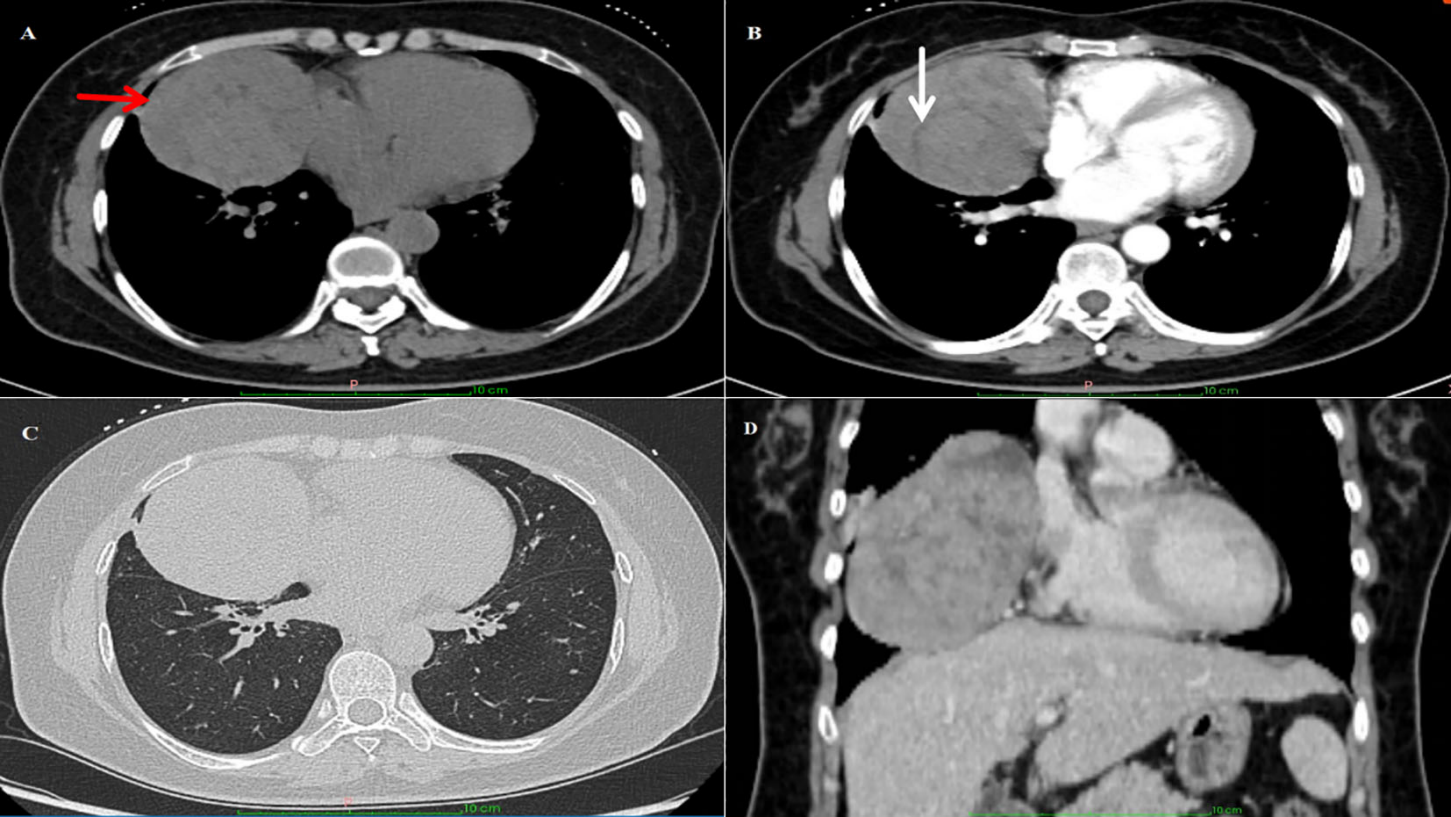
Imaging findings: **(A)** CT image of the chest showing a large irregular mass near the anterior mediastinum of the right thorax. The red arrow highlights the location of the tumor. **(B)** The white arrow marks the linear low-density area within the tumor. **(C, D)** The mass compressed the bronchi in the middle lobe of the right lung, and the right atrium was mildly compressed.

A 49-year-old woman presented with exertional dyspnea had persisted for more than one month, without concomitant symptoms such as fever, cough, or chest pain. Her medical history included an appendectomy performed 20 years ago. Family history was notable for maternal death from lung cancer and fraternal death from hepatocellular carcinoma. Physical examination revealed no remarkable abnormalities. Hematologic analysis revealed leukopenia (leukocyte count: 3.23×10^9^/L; reference range: 3.5-9.5×10^9^/L) and normal tumor markers (CEA: 0.62 ng/mL [<5.00]; CA19-9: 13.50 U/mL [<30]). The endocrine profile showed decreased ACTH (<1.00 ng/L; reference: 5.00-78.00) and decreased renin activity (supine: 0.88 μIU/mL [reference: 2.80-39.90]; upright: <0.50 μIU/mL [reference: 4.40-46.10]). All catecholamine metabolites, including plasma free metanephrines and 24-hour urinary vanillylmandelic acid (VMA), were within normal ranges, with no other clinically significant abnormalities. Contrast-enhanced CT images demonstrated a well-circumscribed, round mass (8.1×7.9 cm) in the right paracardiac mediastinum, showing predominantly soft-tissue attenuation with internal hypodense septations. A markedly hyperenhancing component was observed within the lesion, while another isodense lesion was seen adjacent to the mass with no significant enhancement. The bronchus of the middle lobe of the right lung was compressed, adjacent to the right cardiac border, and the right atrium was compressed (**Figures 1A-D**). No destruction of the sternum or ribs was observed, and no pleural effusion was present.

**Figure 2:**
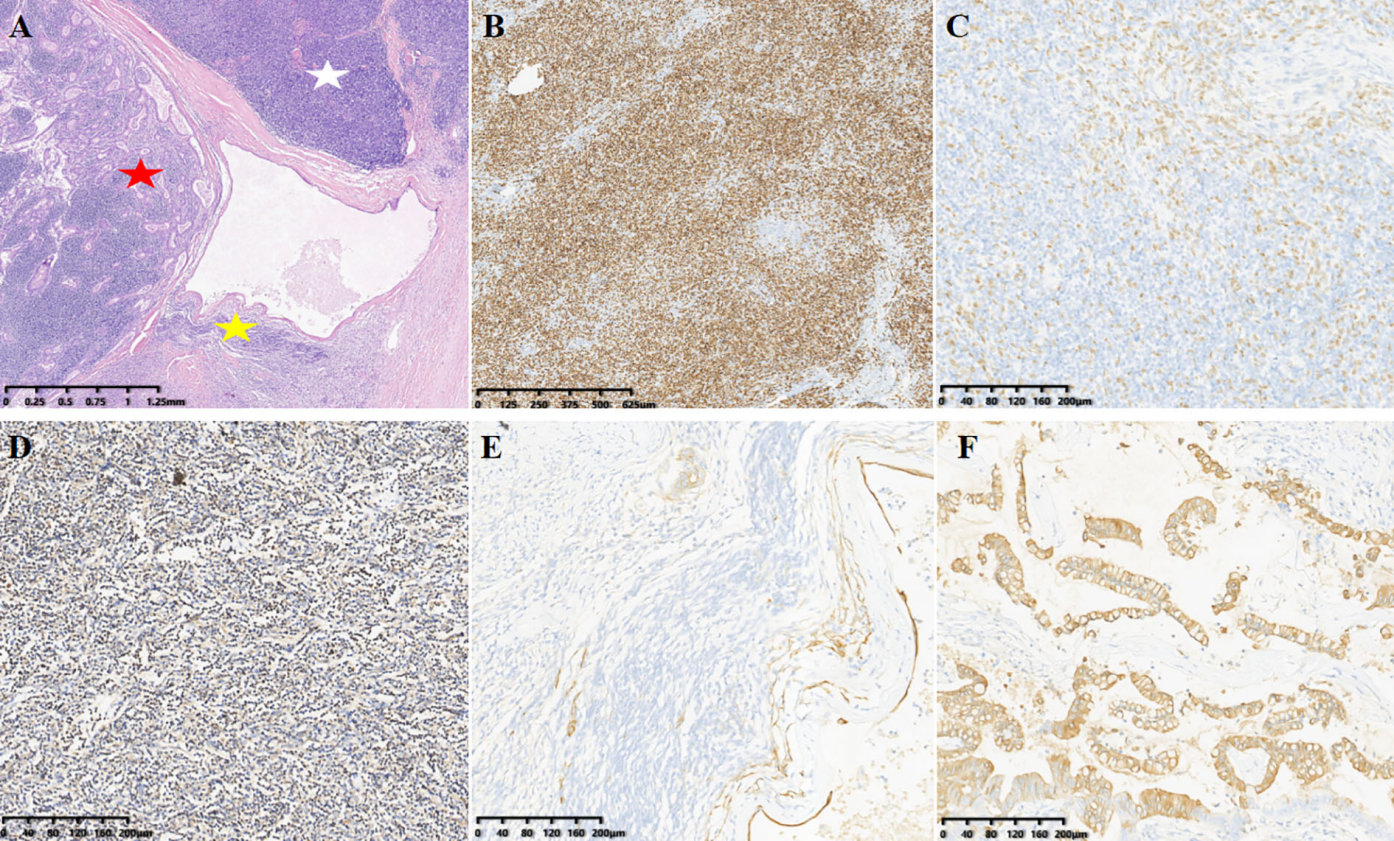
**(A)** (H&E): Magnification, x20. The white star indicates Type B thymoma, the yellow star indicates Type A thymoma, and the red star indicates adenocarcinoma. **(B-F)** (IHC) show specific markers: [**(B–D)**, Type B thymoma)]. **(B)** background lymphocytes TdT(+), magnification, ×40. **(C)** Thymic component p63(+), magnification, ×100. **(D)** CD1a(partially +), magnification, ×100. [**(E)**, Type A thymoma] CK(Pan)(+), magnification, ×100. [**(F)** adenocarcinoma] CK20(focally +), magnification, ×40.

The patient underwent complete mass resection and thymectomy via a right-sided thoracoscopic approach. Grossly, the mass was observed as grayish-white tumor tissue with an intact capsule and firm consistency, located in the right mediastinal plane. Upon sectioning, the tumor demonstrated cystic-solid components containing gelatinous fluid, with smooth inner and outer walls, no adhesion to the adjacent pleura, and no invasion into lung lobes or pericardium. The immunohistochemistry results revealed the following: glandular component CK19 (+), CDX-2 (weakly +), PCK (+), CK7 (small foci +), CK20 (+), negative for SATB2, PAX-2, WT-1, ER, P16, TTF-1, PAX-8, and P53, with Ki-67 index of 5–10%. Thymic component P63 (+), background lymphocytes TdT (+), CD1a (partially +), and CD20 (focally +). No lower mutation peak at codon 424 of exon 15 of the GTF2I gene was not found, and codon 12 and 13 mutations of the KRAS gene were not detected. The final pathological diagnosis was collision tumor, type AB thymoma + well-differentiated adenocarcinoma (likely of malignant foregut cyst origin) (**Figures 2A-D**). Postoperatively, the patient experienced an uneventful recovery without recurrence. The decision to forgo adjuvant chemotherapy/radiotherapy was based on histologically confirmed R0 resection and low-risk pathological features (lymphovascular invasion-negative, pT1N0M0 stage).

## Discussion

Collision tumors are a distinct clinicopathological entity characterized by the coexistence of two or more histologically distinct neoplastic components within a single anatomical site, maintaining their respective tissue origins and juxtaposed without transitional zones or migratory changes (7). Although collision tumors are clinically uncommon, mediastinal cases are exceptionally rare. Previously reported mediastinal collision tumor combinations have included gastric adenocarcinoma and esophageal cancer (2, 19), synovial sarcoma and arteriovenous (AV) malformation (20), embryonal rhabdomyosarcoma and benign teratoma (21), B1B2 thymoma and small lymphocytic lymphoma (22). The present case demonstrates a mediastinal collision tumor composed of type AB thymoma and well-differentiated adenocarcinoma (likely of malignant foregut cyst origin). To the best of our knowledge, this tumor combination has not been previously documented in the anterior mediastinum.

The pathogenetic mechanisms underlying the diverse clinical manifestations and nonspecific imaging features of collision tumors remain incompletely understood. Several pathogenic hypotheses have been proposed for mediastinal collision tumors: First, the tumor microenvironment theory suggests that the development of one tumor may alter the local microenvironment, facilitating the growth of a second primary or metastatic tumor. Second, the accidental concurrence theory proposes that two independent tumors may coincidentally arise at the same anatomical site. Third, the tumor genetics theory postulates that clonal cells with genetic heterogeneity can differentiate into histologically distinct tumor populations (23). Finally, prior trauma, surgical intervention, or radiation therapy has been implicated as potential etiological factors.

The clinical manifestations of mediastinal collision tumors are nonspecific and primarily depend on the tumor location and histological composition. Anterior mediastinal collision tumors typically present with cough (20), chest pain, dyspnea (21), or myasthenia (22). Tumors located at the esophagogastric junction most frequently cause dysphagia, food obstruction sensation, and retrosternal discomfort (2, 19). A proportion of cases remain asymptomatic and are detected incidentally during routine physical examination.

Preoperative evaluation plays a crucial role in treatment planning and prognosis assessment. Contrast-enhanced CT, with its superior spatial and density resolution combined with multiplanar reformation, enables accurate lesion detection and evaluation, thus establishing a reliable basis for surgical strategy formulation. Although CT is widely used as a primary diagnostic tool, its soft-tissue resolution remains limited. Future studies should incorporate multiparametric imaging protocols to improve clinicopathological correlation, particularly by integrating contrast-enhanced MRI for neurovascular invasion assessment along with 18F-FDG PET-CT for metabolic profiling, metastasis surveillance, and TNM staging refinement.

Regarding CT characteristics of thymomas, some studies have reported that type AB thymomas typically demonstrate multiple markedly enhancing nodules, hypodense septations, and intratumoral penetrating vessels (24). Most foregut cysts are predominantly located in the middle and posterior mediastinum, with malignant transformation being exceptionally rare (25). On CT imaging, foregut cysts appear as round or oval masses with smooth margins, showing fluid/soft-tissue density on unenhanced scans without contrast enhancement. In the current case, the anterior mediastinal collision tumor presented as a rounded mass with well-defined borders, heterogeneous attenuation, predominantly showing soft-tissue attenuation, enhanced nodules and hypodense septations, findings consistent with type AB thymoma. Adjacent to this was a second soft-tissue mass that appeared isointense without enhancement, features suggestive of jelly-like fluid content. Although thymomas are the most common anterior mediastinal tumors, foregut cysts in this location remain exceedingly uncommon.

Mediastinal collision tumors exhibit diverse imaging characteristics due to the distinct origins of their tumor components. The diagnosis of mediastinal collision tumor should be considered when there is a marked disparity in boundaries and enhancement patterns between two adjacent tumors, especially when typical imaging features of two different tumor types are present without a unifying pathological explanation. Given the rarity of these tumors, accurate identification of both morphological characteristics and histological components is essential to avoid misdiagnosis. Furthermore, accumulating additional case data and conducting long-term follow-up are crucial for advancing understanding of the disease and assessing treatment outcomes.

A review of the literature combined with this case report has identified that the main features of lesions requiring imaging differentiation from mediastinal collision tumors include: 1) Noninvasive thymoma, is characterized by regular morphology, homogeneous soft-tissue attenuation, absence of mediastinal or vascular invasion, and uniform enhancement; 2) Invasive thymoma, demonstrates irregular contours, heterogeneous attenuation, marked heterogeneous enhancement, extensive cystic degeneration/necrosis, obliteration of mediastinal fat planes, and growth along the pericardial/thymic planes; 3) Non-Hodgkin lymphoma(NHL), presents with smooth margins, homogeneous density, rare calcification, variable enhancement, and small necrotic foci with sharp margins, often invading adjacent structures and accompanied by lymphadenopathy; 4) Teratoma, typically occurs in younger patients, containing calcifications/dental structures, which frequently causes hemoptysis, with well-demarcated borders and heterogeneous attenuation; 5) Yolk sac tumor, predominantly unilocular cystic lesions with ill-defined margins and local invasion; 6) Mediastinal lung carcinoma, shows spiculated margins, lobulation, and with high-grade features at the lung-tumor interface, with homogeneous or heterogeneous enhancement and metastatic propensity; 7) Paraganglioma, exhibits intermediate soft-tissue attenuation with cystic changes, heterogeneous enhancement, intralesional vascularity, mass effect without frank invasion, and requiring confirmation of biochemical catecholamine levels.

In summary, collision tumors in the anterior mediastinum are exceedingly rare, particularly those comprising thymoma and adenocarcinoma. Their clinical and imaging features often overlap with more common tumors in this region, potentially leading to diagnostic oversight. Definitive diagnosis ultimately requires postoperative histopathological examination. Nevertheless, comprehensive imaging evaluation provides essential information regarding tumor location, dimensions, margin characteristics, adjacency, lymph node metastasis and tumor infiltration, thereby delivers critical diagnostic and prognostic information for clinical management and follow-up.

## Data Availability

The datasets presented in this article are not readily available because of ethical and privacy restrictions. Requests to access the datasets should be directed to the corresponding author.
