# Supplementary figures and images for: Anterior mediastinal collision tumor of type AB thymoma and adenocarcinoma: a case report
Source: Front Oncol. 2025 May 16;15:1495627. doi: 10.3389/fonc.2025.1495627 (PMC12122290; doi:10.3389/fonc.2025.1495627)

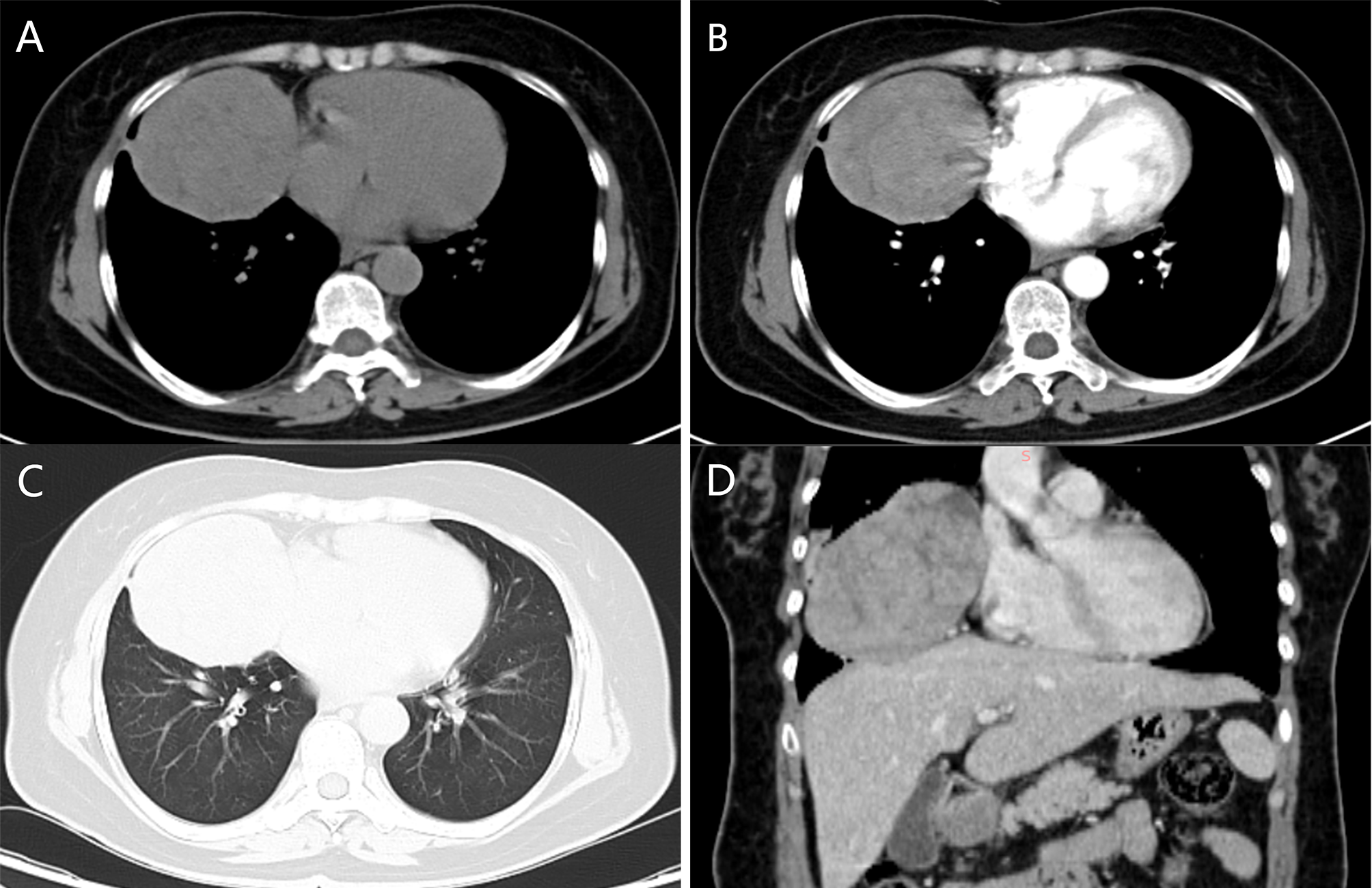

Supplement: Supplementary file 1 [file Image1.tif]

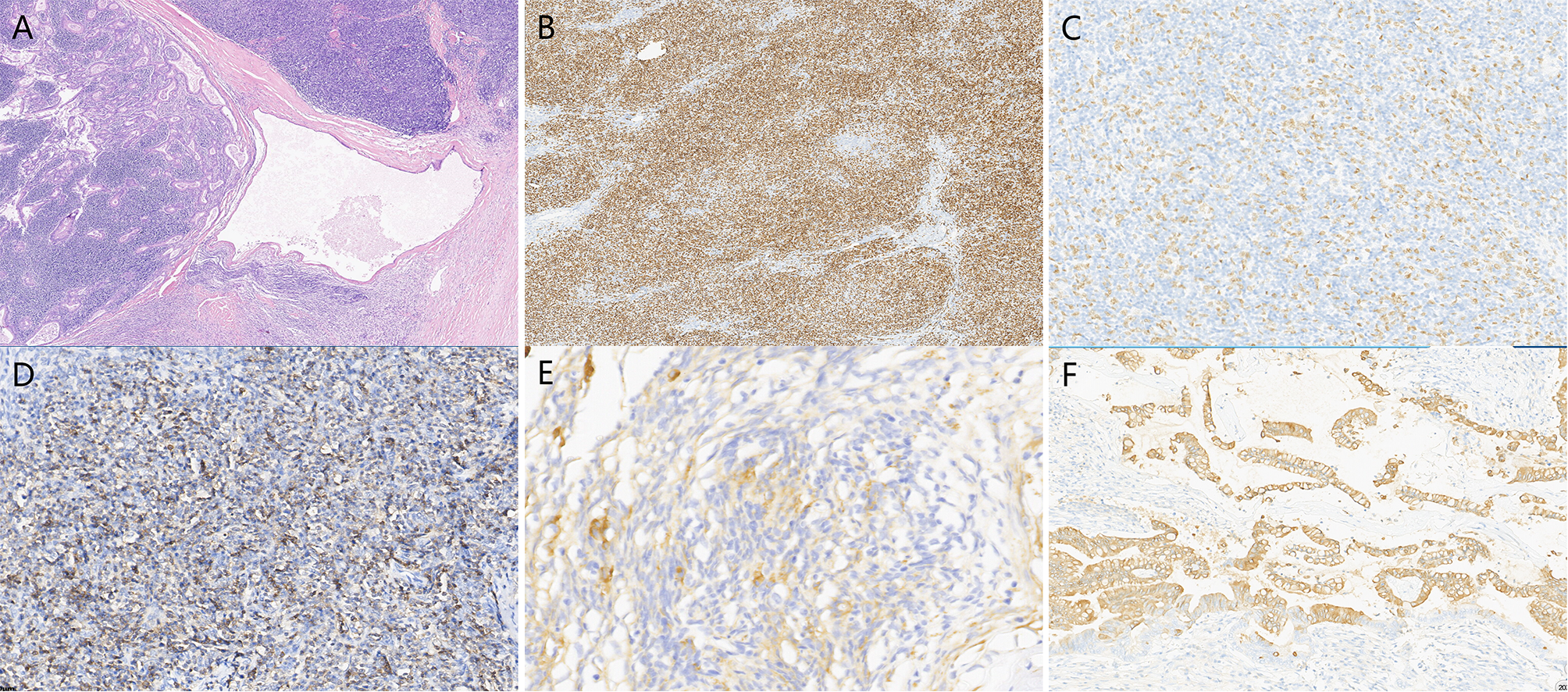

Supplement: Supplementary file 2 [file Image2.tif]
